# Dengue virus-induced ER stress is required for autophagy activation, viral replication, and pathogenesis both *in vitro* and *in vivo*

**DOI:** 10.1038/s41598-017-18909-3

**Published:** 2018-01-11

**Authors:** Ying-Ray Lee, Szu-Han Kuo, Ching-Yen Lin, Po-Jung Fu, Yee-Shin Lin, Trai-Ming Yeh, Hsiao-Sheng Liu

**Affiliations:** 1 Department of Medical Research, Chiayi Christian Hospital, 600 Chiayi, Taiwan; 2 Department of Microbiology and Immunology, College of Medicine, National Cheng Kung University, 701 Tainan, Taiwan; 3 Department of Medical Laboratory, Science and Biotechnology, College of Medicine, National Cheng Kung University, 701 Tainan, Taiwan

## Abstract

Dengue virus (DENV) utilizes the endoplasmic reticulum (ER) for replication and assembling. Accumulation of unfolded proteins in the ER lumen leads to ER stress and unfolded protein response (UPR). Three branches of UPRs temporally modulated DENV infection. Moreover, ER stress can also induce autophagy. DENV infection induces autophagy which plays a promotive role in viral replication has been reported. However, the role of ER stress in DENV-induced autophagy, viral titer, and pathogenesis remain unclear. Here, we reveal that ER stress and its downstream UPRs are indispensable for DENV-induced autophagy in various human cells. We demonstrate that PERK-eIF2α and IRE1α-JNK signaling pathways increased autophagy and viral load after DENV infection. However, ATF6-related pathway showed no effect on autophagy and viral replication. IRE1α-JNK downstream molecule Bcl-2 was phosphorylated by activated JNK and dissociated from Beclin 1, which playing a critical role in autophagy activation. These findings were confirmed as decreased viral titer, attenuated disease symptoms, and prolonged survival rate in the presence of JNK inhibitor *in vivo*. In summary, we are the first to reveal that DENV2-induced ER stress increases autophagy activity, DENV replication, and pathogenesis through two UPR signaling pathways both *in vitro* and *in vivo*.

## Introduction

DENV contains four serotypes (DENV1 to DENV4). DENV infection may cause diseases from mild dengue fever to severe syndromes of dengue shock syndrome (DSS) and dengue hemorrhagic fever (DHF). DHF is characterized by increased vascular permeability, hypovolemia and abnormal blood clotting mechanisms. However, the mechanism of DHF is not fully understood. Viral load and chemical mediators including cytokines are the possible regulators involved in the pathogenesis of DHF and DSS^1–3^.

Previous studies demonstrated that flaviviruses are endoplasmic reticulum (ER) tropic, which target the host ER for their replication^4^. ER participates in various biological functions including lipid synthesis, Ca^2+^ ion homeostasis, protein folding and maturation, and is the major signal transduction organelle that responds to the alterations. Accumulation of unfolded protein or viral infection disrupted the balance of ER to cause ER stress^5,6^. Prolonged ER stress leads to cell death through apoptosis or autophagy^7^. Three distinct pathways including Protein kinase-like endoplasmic reticulum kinase (PERK), inositol-requiring protein-1α (IRE1α), and activating transcription factor-6 (ATF6) have been reported to be the downstream mediators of UPR signaling under ER stress. These pathways are inactivated by binding with the chaperone protein GRP78/Bip under normal conditions. Accumulation of unfolded proteins in ER lumen triggers dissociation of GRP78/Bip from the sensor and activates the three signaling pathways^6^. First, PERK signaling pathway is induced by various stressors. Activated PERK phosphorylates eIF2 at Ser51 of α subunit to block its function and attenuates protein synthesis to prevent further unfolded protein accumulation. Activating transcription factor 4 (ATF4) is the linker between PERK and apoptosis or autophagy process, and is essential for the up-regulation of many ER stress-related genes including autophagy related protein (ATG12) and CHOP that participates in autophagy induction^8,9^. Second, IRE1α, a yeast Ire1 gene product, splices the X-box binding protein 1 (XBP1) mRNA and converts the spliced sXBP1 into a transcriptional activator to induce UPR genes including chaperon and ER-associated degradation (ERAD). Moreover, IRE1α phosphorylates c-Jun N-terminal kinases (JNK) which regulates both autophagy and apoptosis activities through the regulation of Bcl-2 family proteins^10,11^. Third, ATF6 dissociates from GRP78/Bip during ER stress and translocates to Golgi apparatus where it is cleaved by site-1 (SP1) and site-2 (SP2) proteases. Cleaved ATF6 then enters the nucleus and promotes the ER chaperone gene transcription^12^. Virus infection targets ER to sustain its replication. However, prolonged ER stress induces autophagy or apoptosis process. The regulations of UPR pathways by flaviviruses have been reported, including West Nile virus (WNV), Japanese encephalitis virus (JEV) and DENV^13–15^. UPR modulates the production of various cytokines or chemokines including interleukin-6 (IL-6), interleukin-8 (IL-8), and tumor necrosis factor (TNFα), which are involved in DHF and DSS pathogenesis^16,17^. There are no reports that link the UPR, autophagy and cytokines production under DENV2 infection.

Autophagy is a self-degradative process to maintain cellular homeostasis, and is implicated in diverse pathophysiological processes including infectious diseases^18,19^. DENV, Hepatitis C virus (HCV) and JEV infection increases autophagic activity to promote viral replication^20–23^. DENV NS4A gene induces PI3K-dependent autophagy and protects cells from death^24^. Further studies reveal that DENV and HCV utilize autophagic degradation machinery to subvert host cell lipid system and promote their production^25,26^. Autophagy and autophagy-related genes have anti-viral or pro-viral functions of multiple viruses^27,28^. Autophagy suppresses interferon (IFN) and inhibits inflammasome-dependent maturation and secretion of interleukin-1β (IL-1β) and interleukin-18 (IL-18) to the invading pathogens^29–31^. Accumulating evidence shows that autophagy participates in protein trafficking and secretion^32^. Altogether, autophagy is responsible for multiple cell mechanisms including promotion of viral replication, regulation lipid metabolism and immune responses.

Molecules involved in UPR also participate in activation of autophagy signaling pathways^33,34^. HCV infection or overexpression of individual HCV proteins in the ER compartment induces ER stress and associated UPR^35^. However, their findings are contradictory^36,37^. One report claimed that HCV-induced UPR regulates viral replication through autophagy^38^. DENV infection could also induce ER stress and autophagy^15,22,23,25,39^. However, the underlying mechanism of how ER stress affects autophagy remains unclear. In this study, we investigate the pathways and the mechanisms between DENV-triggered ER stress and autophagy as well as the effects on viral replication and pathogenesis *in vivo*.

## Results

### DENV2-induced ER stress affects autophagic activity and viral replication

**Figure 1:**
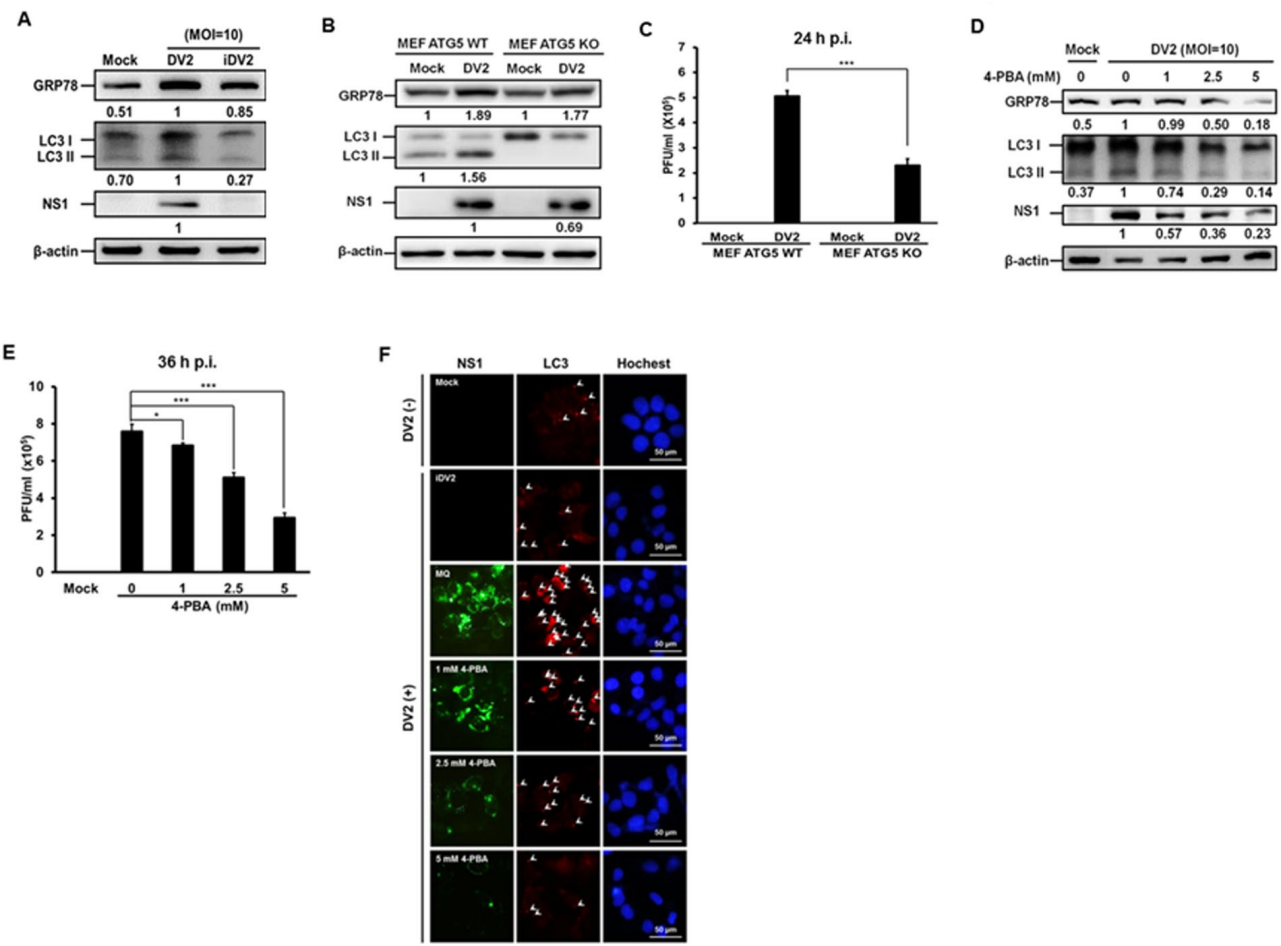
DENV2 infection triggers autophagy and increases viral titer through activation of ER stress. (**A**) Huh7 cells were infected with either 10 MOI of DENV2 or iDENV2 and the protein levels of GRP78, LC3-II/ LC3-I and NS1 were determined by Western blotting at 36 h p.i. (**B**) MEF-Atg5 wild-type (MEF ATG5 WT) and knockout cells (MEF ATG5 KO) were infected with DENV2 (MOI = 20). The proteins were examined by Western blotting at 24 h p.i. (**C**) Viral titer of (**B**) was determined by plaque assay. (**D**) Huh7 cells were infected with DENV2 (MOI = 10) and treated with or without 4-PBA at different doses and the proteins were determined by Western blotting at 36 h p.i. β-actin was used as an internal control. The number below the band is the quantification of band intensity after normalization with β-actin. (**E**) Viral titer of (**D**) was determined by plaque assay. (**F**) Under the same conditions as (**D**), the FITC-labeled DENV2 NS1 (green), PE-labeled LC3 puncta (red) and Hochest labeled nuclei (blue) were detected under the fluorescent microscopy. Mock means no infection with DENV2, DV2 means DENV2 infection, and iDV2 means heat inactive DENV2.

Previous studies have shown that DENV2 infection induces both ER stress and autophagy^15,23,25,39–41^. DENV-induced ER stress regulating autophagy in the MDCK (Madin Darby canine kidney) cells has been reported^39^. We previously reported that DENV2 induced autophagy enhances viral replication both *in vitro* and *in vivo* ^22,23^. Here we clarified the relationship among DENV induced ER stress, autophagy activation and viral replication. Initially, Huh7 cells were infected with live or heat-inactivated DENV2 (DENV2 and iDENV2) at a MOI of 10 and the samples were collected at 36 h p.i. to clarify whether DENV2 infection induced both ER stress and autophagy *in vitro*. Figure 1A shows that DENV2 infection increased the expression of GRP78 (an indicator or ER stress) and LC3-II protein (an indicator of autophagy activity) compared to mock and iDENV2 infection, indicating that live DENV could induce ER stress and autophagy. Compared to DENV2 infection, only ER stress but not autophagy was mildly induced by iDENV2, suggesting that only live DENV2 could induce ER stress and autophagy in Huh7 cells. To determine the relationship between autophagy and DENV2-mediated ER stress, the wild type *ATG5* gene (an essential gene of autophagy) mouse embryo fibroblast (MEF ATG5 WT) and knockout (MEF ATG5 KO) cells were used. We found that knockout of *ATG5* gene showed no effect on DENV2 infection induced GRP78 expression (Fig. 1B), suggesting that autophagy progression is not at the up-stream of DENV2-induced ER stress. Similarly, above finding was observed in DENV2 infected MEF ATG5 wild type and ATG5 silencing cells (S1 appendix). However, knockout of *ATG5* gene decreased NS1 protein expression as well as viral titer in DENV infected Huh 7 cells compared to DENV infected wild type MEF cells (Fig. 1B, lane 2 vs. lane 4 and Fig. 1C). We further used the ER stress inhibitor 4-PBA (4-phenyl butyric acid) to clarify whether ER stress affects DENV2-triggered autophagic activity. Huh7 cells were treated with 4-PBA at various concentrations after DENV2 infection, the expressions of GRP78, LC3-II as well as DENV2 NS1 were examined at 36 h p.i. The result showed that blocking ER stress decreased the levels of GRP78, LC3-II as well as viral replication demonstrated by Western blotting and plaque assay (Fig. 1D and E). The inhibitory effect of 4-PBA on autophagic activity during DENV2 infection was further confirmed by immunofluorescence assay. Our data show that the LC3 puncta and the NS1 expression level decreased by the treatment of 4-PBA in a dose dependent manner in DENV2 infected Huh 7 cells (Fig. 1F). Altogether, we demonstrate that DENV2-induced ER stress is at the up-stream of autophagy to regulate viral replication.

### Induction of UPR by DENV2 infection is a general event in various cell lines

**Figure 2:**
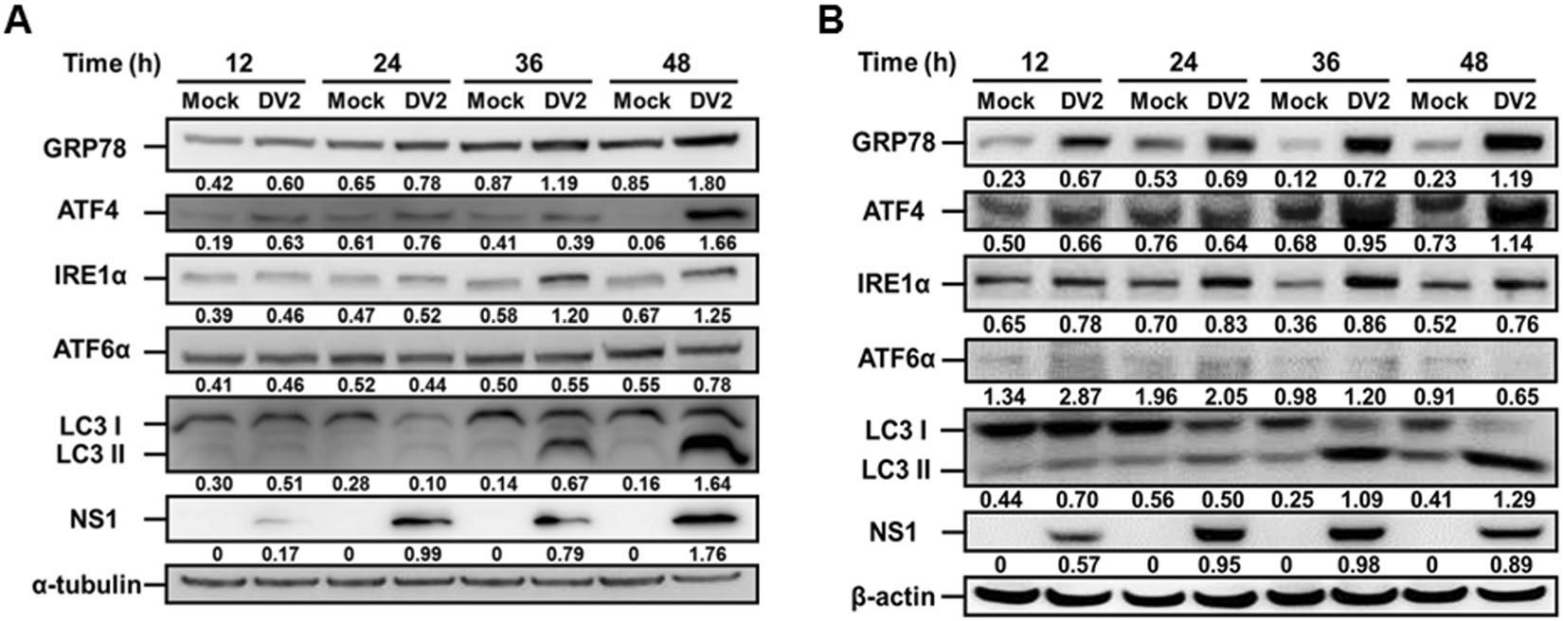
Modulation of UPR pathways during DENV2 infection is a general event in various human cell lines. (**A**) Hepatoma Huh7 cells and (**B**) lung cancer A549 cells were infected with DENV2 (MOI = 10) and the ER stress-related markers including GRP78, ATF4, IRE1α and ATF6α as well as the autophagy marker LC3-II/LC3-I and DENV2 NS1 protein expression was evaluated by Western blotting using the specific antibodies. During ATF6α activation, it is cleaved from the total form (90 kDa) to the active form (50 kDa). The anti-ATF6α antibody used in this study can detect the total form ATF6α only. The number under the band is the quantification of band intensity after normalization with β-actin.

Previous studies reported that DENV infection of two unconventional cell lines (human fibrosacoma 2fTGH and MDCK cells) induced UPR in a time-dependent manner^15,39^. To clarify whether DENV modulated UPR regulates autophagic activity in the generally used cell lines, human hepatoma Huh7 and lung cancer A549 cells were infected with DENV2 at the MOI of 10, and the cell lysates were collected at 12 h, 24 h, 36 h and 48 h p.i. The expression levels of GRP78 (a marker of ER stress), ATF4 (a marker of PERK pathway), IRE1, ATF6, LC3 II (autophagy marker) as well as NS1 were investigated during DENV2 infection. We found that the levels of GRP78, ATF4, and IRE1α started to increase at 12 h p.i. and consistently raised at 36 h and 48 h p.i. in both Huh7 (Fig. 2A) and A549 cells (Fig. 2B). Furthermore, autophagy marker LC3-II together with DENV2 NS1 expression increased at 36 h and 48 h p.i. However, ATF6 expression fluctuated from 12 h to 48 h p.i. in both of the cell lines (Fig. 2A and B). These data imply that among the ER stress regulated UPRs, PERK and IRE1 signaling pathways may play a dominant role in DENV2-induced autophagy, and ATF6 is not a major pathway involved. These findings could be detected in various human cell lines.

### PERK and IRE1 signaling pathways are involved in DENV2-induced autophagy and viral replication

**Figure 3:**
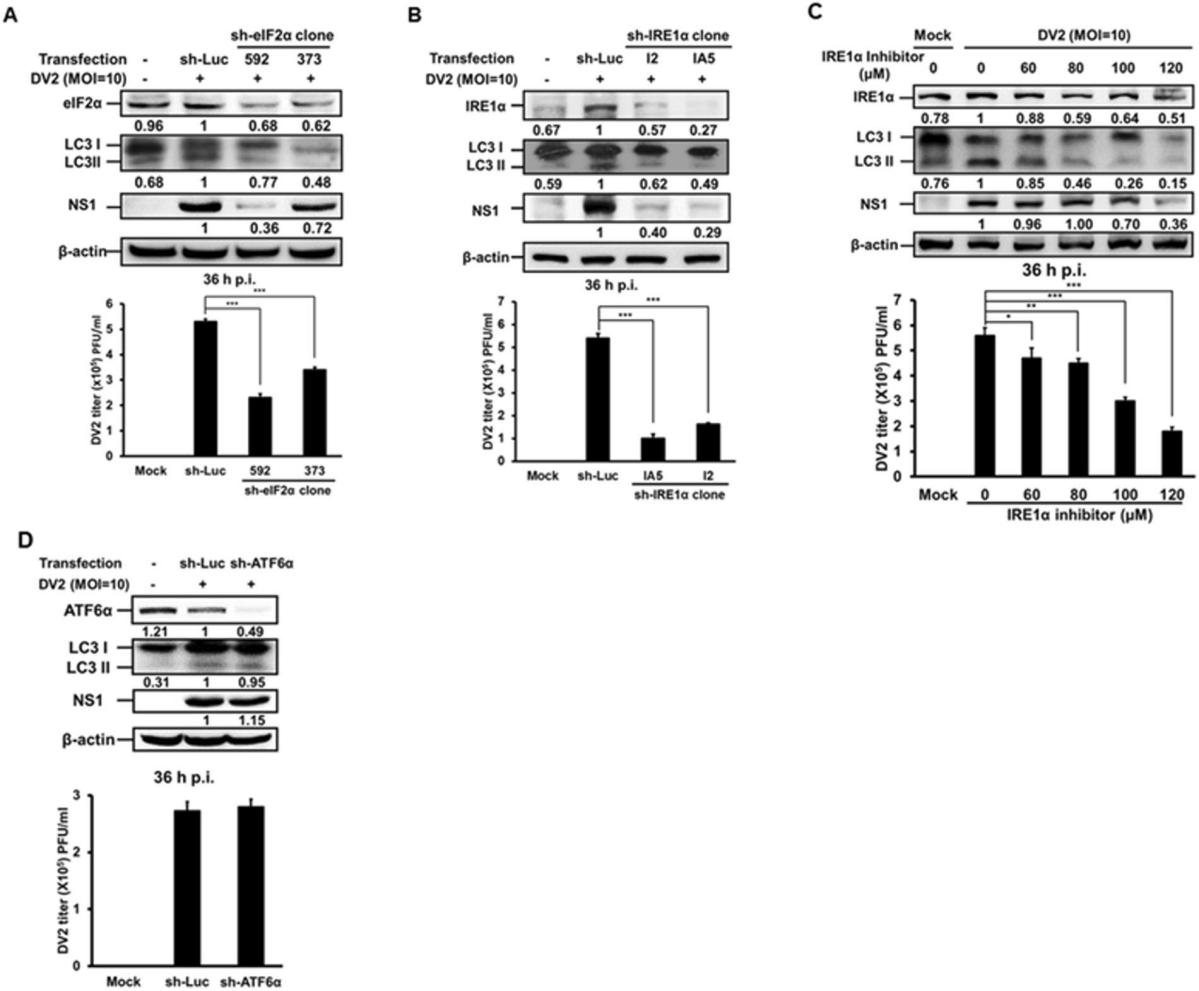
PERK and IRE1 signaling pathways are involved in DENV2-induced autophagy activation. (**A**) Huh7 cells were transfected by Turbofect^™^ with sh-eIF2α for 18 h and infected with DENV2 (MOI = 10). (**B**) Huh7 cells were transfected with sh-IRE1α for 18 h and infected with DENV2 (MOI = 10). (**C**) Huh7 cells were infected with DENV2 (MOI = 10) followed by IRE1α inhibitor treatment at different concentrations. (**D**) Huh7 cells were transfected with sh-ATF6α for 18 h and infected with DENV2 (MOI = 10). The protein levels of eIF2α, IRE1α and ATF6α were measured by Western blotting at 36 h p.i. The number under each band is the quantification of band intensity after normalizationwith β-actin. The viral titers were determined by plaque assay.

Above findings indicate that DENV2 induced ER stress and PERK and IRE1 two signaling pathways are the up-stream regulator of autophagy. To confirm this speculation, Huh7 cells were transiently transfected with the specific sh-RNAs against eIF2α (PERK pathway) and IRE1α followed by DENV2 infection, and the expression of eIF2α and IRE1α was determined by Western blotting. We reveal that eIF2α and IRE1α expression levels were suppressed to various degrees, which correlate with decreased levels of DENV2 NS1 and autophagy LC3-II protein expression as well as viral titer (Fig. 3A,B and quantified diagrams). Figure 2A and B showed that among the three UPR signaling pathways, IRE1α pathway was most significantly induced by DENV2 compared to the other two pathways at 36 h p.i. At this time point, autophagic activity was also significantly induced. Therefore, the following studies focused on IRE1 signaling pathway. DENV2-infected Huh7 cells were treated with or without 3,5-Dibromosalicylaldehyde (an IRE1α inhibitor). Our data disclosed that IRE1α and DENV2 NS1 expression dose-dependently decreased accompanied with decreased LC3-II expression and viral titer (Fig. 3C and quantified diagram). However, silencing of ATF6α showed decreasing ATF6α protein expression but no effect on LC3-II, NS1 protein expression and viral titer, indicating no influence on autophagy activity and viral replication (Fig. 3D and quantified diagram). In summary, our data suggest that PERK and IRE1 are the major signaling pathways involved in DENV2-induced autophagy and increased viral titer.

### PERK-eIF2α-ATF4-ATG12 signaling pathway participates in early stage of DENV2-induced autophagy and increased viral titer

Xiao reported that ATG5-ATG12 complex is responsible for the initiation of autophagic progression and helps autophagosome formation^42^. Kouroku *et al*. reported that PERK-eIF2α-ATG12 signaling pathway participates in LC3 conversion while polyglutamine 72 repeat (polyQ72) aggregates induce ER stress-mediated cell death with caspase-12 activation and vesicular formation (autophagy)^8^. To clarify whether PERK-eIF2α-ATG12 signaling pathway was activated, the expression levels of GRP78, LC3-II, DENV2 NS1, PERK downstream phosphorylated eIF2α, ATF4 and ATG12 were measured in DENV2 infected Huh7 cells. Our result showed that DENV2 NS1 protein was detectable at 6 h p.i. However, the levels of GRP78 and LC3-II did not increase until 24 h p.i. (S2 appendix). The expression levels of phosphorylated eIF2α, ATF4, free form ATG12, and conjugated ATG12 were up-regulated at 6 h p.i., however, the levels of these proteins decreased at 24 h p.i. (S2 appendix). In summary, this data indicates that PERK-eIF2α-ATG12 signaling pathway is transiently activated at the early stage of DENV2 infection and has trivial effect on autophagy.

### IRE1α-JNK-BECN1 signaling pathway participates in DENV2-induced autophagy and increased viral replication

**Figure 4:**
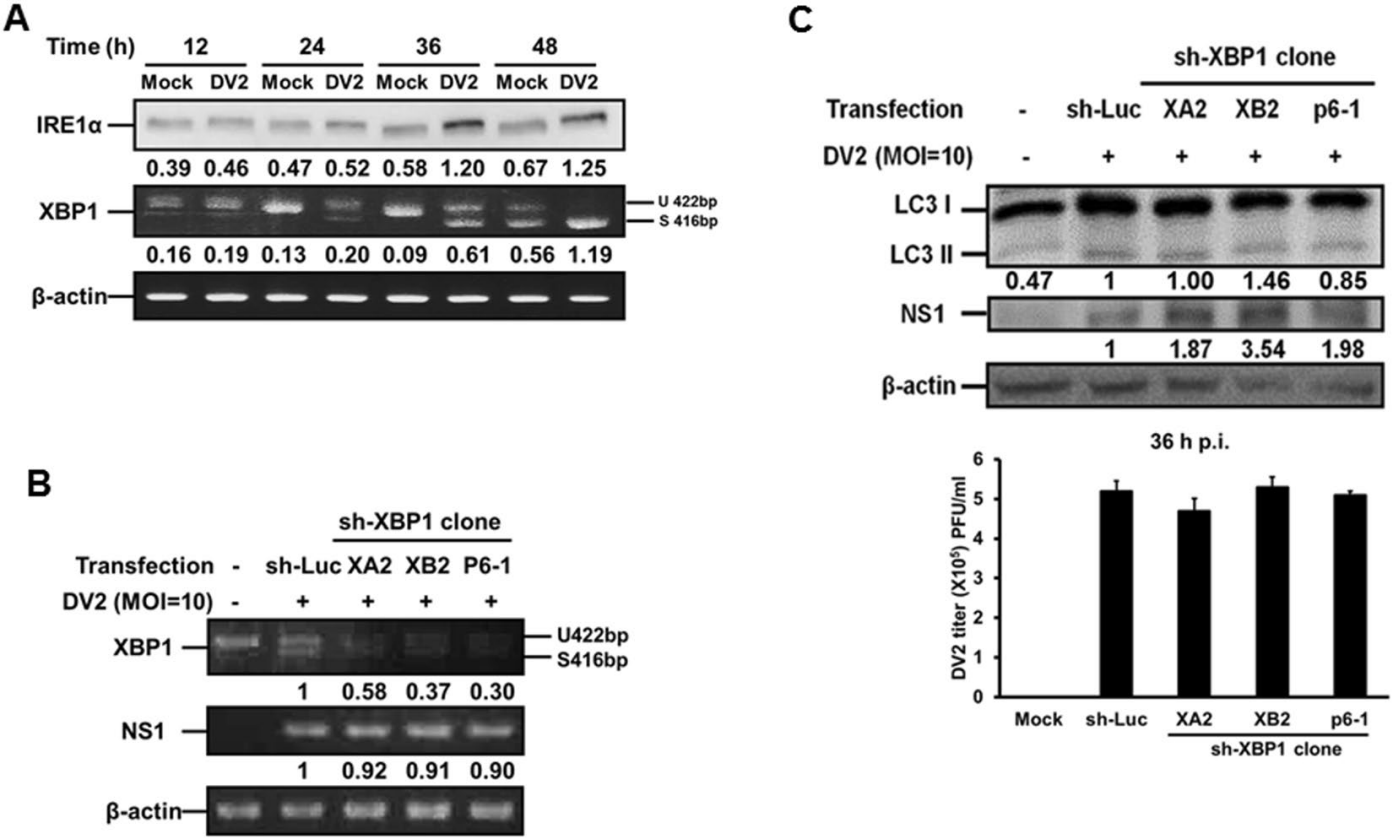
Silencing of XBP1 showed no effect on autophagy activity and viral titer. (**A**) Huh7 cells were infected with DENV2 (MOI = 10). The IRE1α protein level and XBP1 mRNA level were detected at the indicated time points. (**B**) Huh7 cells were transfected with sh-XBP1 for 18 h followed by DENV2 (MOI = 10) infection. The cells were collected at 36 h p.i. (**C**) The condition was as the same as in (**B**). The size of the non-spliced XBP1 is 422 bp and the spliced form is 416 bp. The protein levels of IRE1α, LC3-II/ LC3-I and NS1 were evaluated at 36 h p.i. The number under the band is the quantification of band intensity after normalization with β-actin. The viral titer was determined by plaque assay.

The IRE1α regulates downstream XBP1 and JNK-BECN1 two signaling pathways of UPR. For the former pathway, our result showed that DENV2 infection led to the increase of IRE1α protein expression and cleavage of XBP1 mRNA from 24 h p.i. in DENV2 infected Huh7 cells (Fig. 4A). Furthermore, XBP1 gene silenced by sh-XBP1 in three clones showed decreased XBP1 mRNA expression (both un-cleaved and cleaved forms), but no significant effect on DENV2 NS1 mRNA expression (Fig. 4B). Furthermore, in these three XBP1 silenced clones, LC3-II expression and viral titers were not affected in the presence of DENV2 (Fig. 4C and quantified diagram). In addition, we compared the expression of interleukine-8 (IL-8) and tumor necrosis factor α (TNFα) in the cells with silenced XBP1 gene to that in the cells with normal XBP1 gene expression during DENV2 infection. The data showed that silencing of XBP1 decreased the expression of IL-8 and TNFα in DENV2 infected Huh7 cells (S3A and 3B appendix). In summary, these results suggest that DENV2 infection of Huh7 cells activated IRE1α-XBP1 signaling pathway, which is responsible for the expression of IL-8 and TNFα; however, this signaling axis is not involved in DENV2-mediated autophagy activation and increased viral titer.

**Figure 5:**
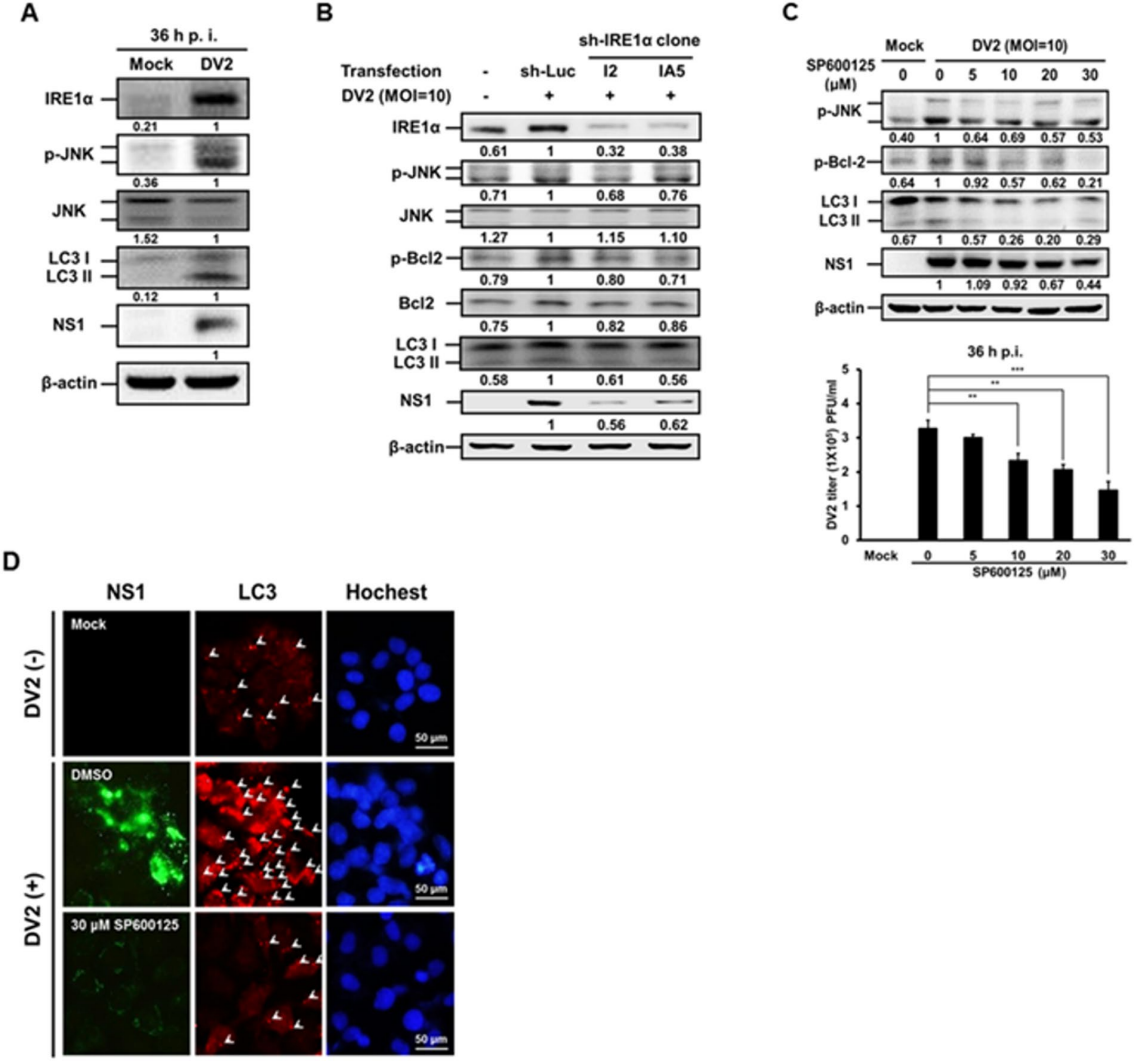
IRE1α-JNK signaling pathway is involved in DENV2-induced autophagy and affects viral titer. (**A**) Huh7 cells were infected with DENV2 (MOI = 10) and the cells were collected at 36 h p.i. (**B**) Huh7 cells were transfected with sh-IRE1α for 18 h and infected with DENV2 (MOI = 10). (**C**) Huh7 cells were infected with DENV2 (MOI = 10) followed by SP600125 treatment at different concentrations. (**D**) Huh7 cells were infected with DENV2 then treated with or without SP600125 (30 μM), and the cells were fixed at 36 h p.i. The FITC-labeled DENV2 NS1 (green), PE-labeled LC3 puncta (red) and Hochest labeled nuclei (blue) were detected under the fluorescent microscopy. Arrow head points the LC3 puncta. The protein levels of IRE1α, p-JNK, JNK, p-Bcl-2, Bcl-2, LC3-II/ LC3-I and NS1 were determined by Western blotting at 36 h p.i. The number under the band is the quantification of band intensity after normalization with β-actin. The viral titer was determined by plaque assay.

For the IRE1α-JNK-BECN1 signaling pathway, JNK (c-Jun N-terminal kinase) belongs to the mitogen-activated protein kinase family, and is response for stress induced apoptosis and autophagy^11^. Here, we demonstrate that both IRE1α and phosphorylated JNK (p-JNK) were up-regulated accompanied with increased expression of LC3-II and DENV2 NS1 proteins in DENV2 infected Huh7 cells at 36 h p.i. (Fig. 5A). However, the expression of JNK total protein did not increase by DENV2 infection (Fig. 5A). We then used sh-IRE1α to suppress IRE1α expression, and found that in two IRE1α silenced clones, decreased expression of IRE1α, p-JNK accompanied with decreased expression of Bcl-2, phosphorylated Bcl-2 (p-Bcl-2), LC3-II and DENV2-NS1 proteins were detected in DENV2 infected Huh7 cells (Fig. 5B). It suggests that IRE1α is an up-stream regulator of JNK, Bcl-2 as well as autophagic activity and viral replication. To validate above interpretation, SP600125, a JNK inhibitor was used. Our data showed that SP600125 decreased the level of p-JNK and led to the reduction of p-Bcl-2, LC3-II, NS1 as well as viral titer (Fig. 5C and quantified viral titer). Moreover, SP600125 inhibitor reduced the number of LC3 puncta and the level of DENV2-NS1 protein in the infected Huh7 cells by immunofluorescent analysis (Fig. 5D). Altogether, above data imply that DENV2 infection elevates the phosphorylation of both JNK and Bcl-2 through activated IRE1α (Fig. 5).

**Figure 6:**
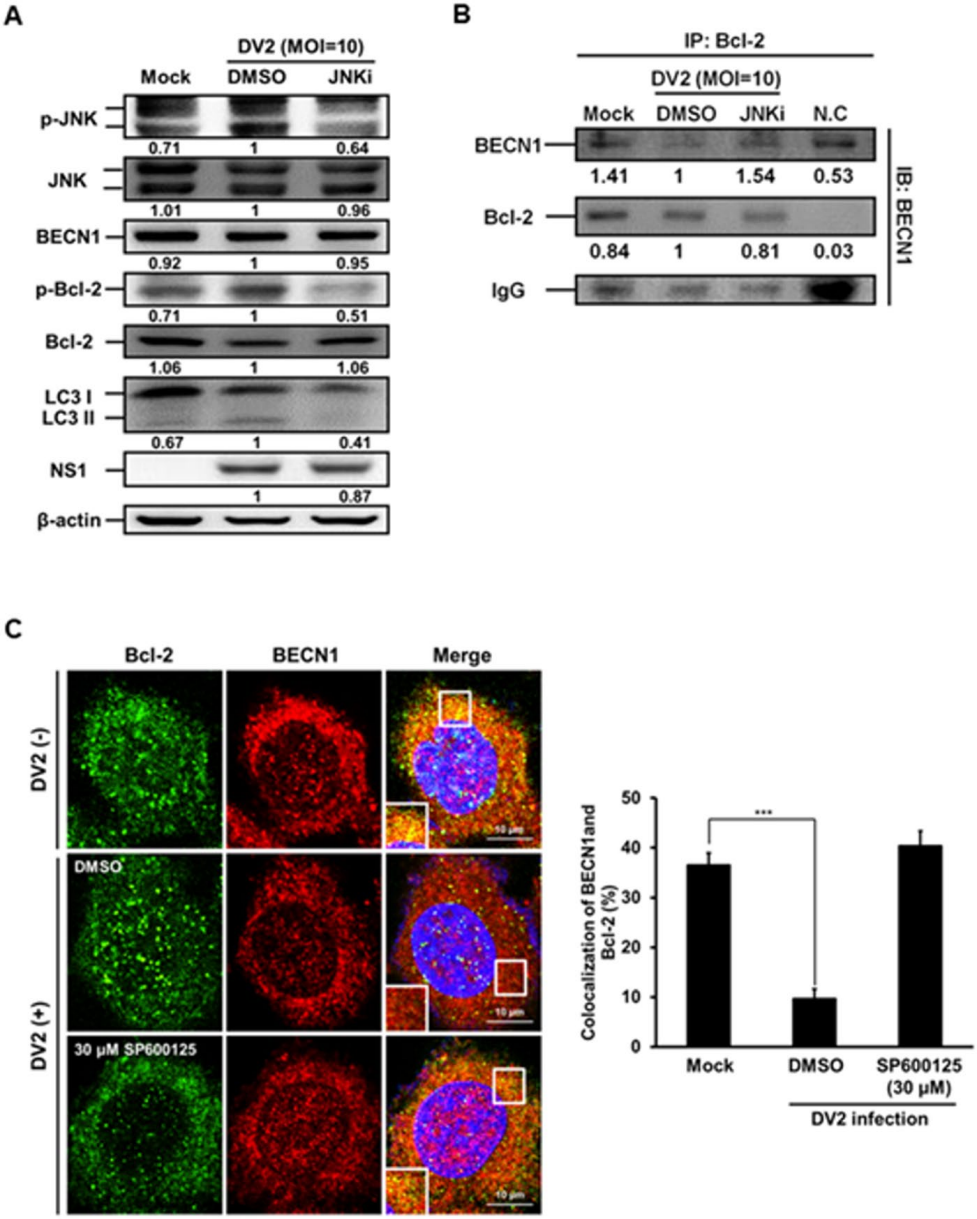
The phosphorylated JNK leads to decreased interaction between Bcl-2 and BECN1 during DENV2 infection, and the effect was rescued by SP600125 treatment. (**A**) Huh7 cells were infected with DENV2 (MOI = 10) with or without the treatment of JNKi-SP600125 (30 μM), and the cells were collected at 36 h p.i. The protein levels of p-JNK, JNK, BECN1, p-Bcl-2, Bcl-2, LC3-II/ LC3-I and NS1 were determined by Western blotting. (**B**) The mouse anti-Bcl-2 antibody (2 μg/ml) was used as the primary antibody to pull-down Bcl-2 protein and incubated with BECN1 antibody at 4 °C for overnight followed by Western blotting to detect the expression of BECN1, Bcl-2 and IgG. The number under the band is the quantification of band intensity and normalized with β-actin. (**C**) Huh7 cells were infected with DENV2 and treated with SP600125 (30 μM), then the cells were fixed at 36 h p.i. The FITC-labeled Bcl-2 and PE-labeled BECN1 were detected under the confocal microscopy. The relative percentage of colocalization of Bcl-2 and BECN1 during DENV2 infection with or without SP600125 treatment was determined.

Our data showed that the levels of p-JNK, p-Bcl-2, LC3-II and DENV2 NS1 proteins evidently increased, but the expression levels of Bcl-2 and BECN1 were not significantly changed at 36 h post DENV2 infection (Fig. 6A, lane 1 vs. lane 2). It is known that Bcl-2 interacts with BECN1^43^. We then clarified the interaction between Bcl-2 and BECN1 by immunoprecipitation to pull down Bcl-2 followed by immunoblotting with anti-BECN1 and anti-Bcl-2 antibodies. We disclosed that the interaction level of BECN1 decreased at 36 h p.i. (Fig. 6B, lane 1 vs. lane 2), indicating decreased interaction between Bcl-2 and BECN1. Furthermore, this phenomenon was reversed in DENV2 infected cells when JNK pathway was blocked by JNK inhibitor (JNKi) (Fig. 6B, lane 2 vs. lane 3). The interaction of Bcl-2 and BECN1 in the presence and absence of DENV2 was further verified by co-localization of these two molecules under the confocal microscope. Our data showed that the colocalization of Bcl-2 and BECN1 was decreased in the presence of DENV2 and this phenomenon was rescued by the treatment of JNK inhibitor SP600125 (Fig. 6C). Altogether, our results imply that IRE1α-JNK-BECN1 pathway is involved in DENV2-induced autophagy and affects viral replication.

**Figure 7:**
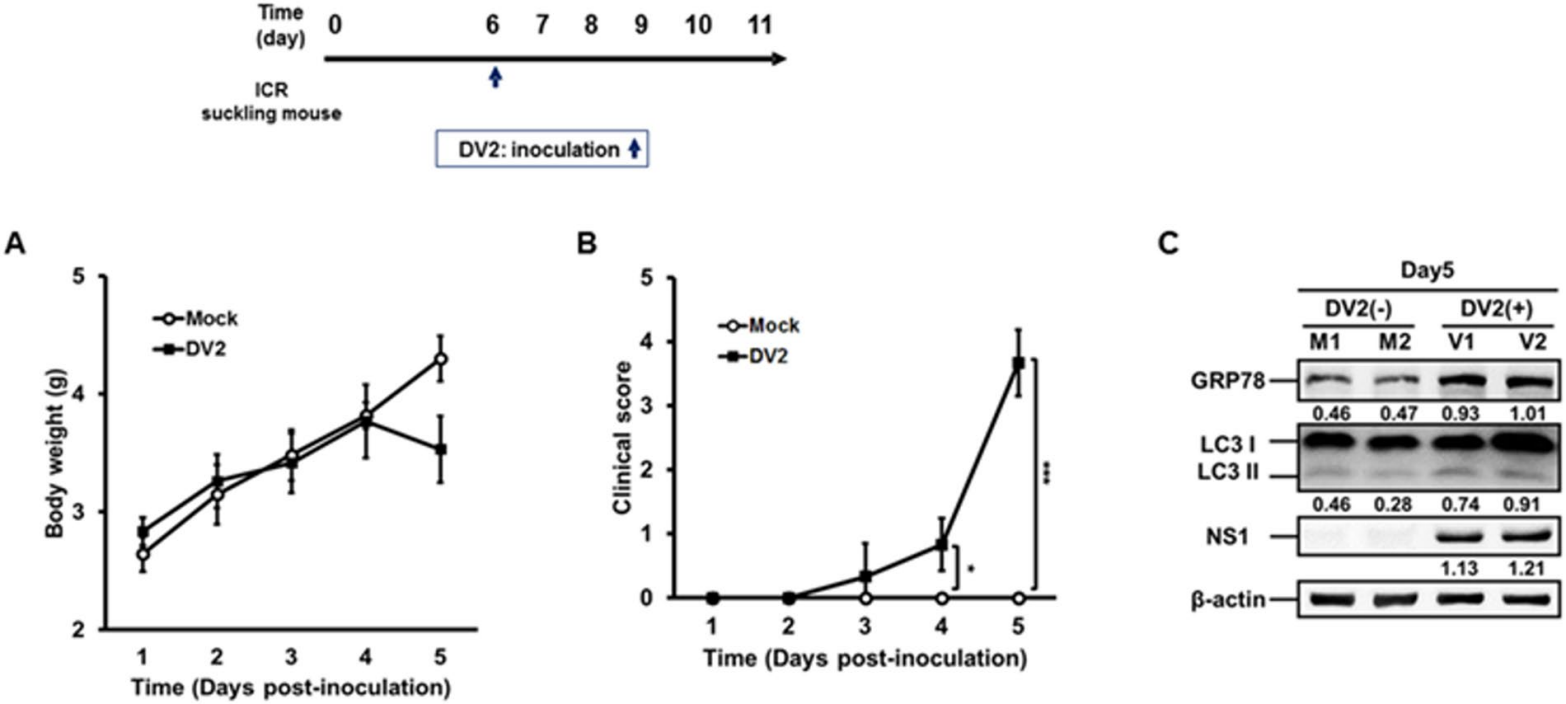
DENV2 infection induces both ER stress and autophagy in ICR suckling mice. Six-day-old ICR suckling mice (Mock n = 6, DENV2 n = 6) were i.c. inoculated with DENV2 (2.5 × 10^5^ PFU/mice). The (**A**) body weight, and (**B**) clinical score were monitored every day after inoculation until day 5. Disease symptoms were scored as following: 0 for healthy. 1 for lightly sick (losing weight and ruffled hair). 2 for moving slowly and reduced mobility. 3 for moving difficulty, forelimb or hindlimb weakness, 4 for paralysis and mortally ill. 5 for death. Mock group was given equal volume of 2% FBS/DMEM. (**C**) The mice were sacrificed at day 5 p.i. The expression of GRP78, LC3-II/LC3-I and NS1 was determined by Western blotting. The number under the band is the quantification of band intensity after normalization with β-actin. M: without DENV2 infection; V: with DENV2 infection.

We further investigated DENV2-induced ER stress associated UPR and autophagy in the human neuroblastoma cells (SK-N-SH). We found that DENV2 permissively infected SK-N-SH cells and increased the expression of GRP78 and LC3-II, indicating increased ER stress and autophagic activity (S4 appendix, lane 2 vs. lane 1). Furthermore, treatment with ER stress inhibitor 4-PBA leads to decreased GRP78, LC3-II and DENV2 NS1 expression in DENV2 infected SK-N-SH cells compared to other two groups (S4 appendix, lane 3). This result is consistent with the data of Huh7 cells (Fig. 1D). We then investigated the effect of DENV2 infection on ER stress, autophagy, viral titer, pathogenesis in the brain of the mouse. Six-day-old ICR suckling mice were intracranially (i.c) inoculated with or without DENV2, and body weight and clinical score were measured every day until day 5 p.i. (Fig. 7). We found that the body weight of DENV2 infected mice significantly decreased at day 5 p.i. (Fig. 7A). Furthermore, DENV2 infected mice showed disease symptoms from day 3 p.i. and these mice were sacrificed at day 5 p.i. (Fig. 7B). The expression levels of GRP78, LC3 and DENV-NS1 proteins in the brain tissues of two mice were shown by Western blotting. We disclosed that the expression of GRP78, LC3-II and NS1 proteins increased in the brains of these two infected mice at day 5 p.i. (Fig. 7C). Altogether, our data imply that DENV2 infection could induce both ER stress and autophagy both *in vitro* and *in vivo*.

### Blocking JNK activation reduces DENV2-mediated autophagy, viral titer, decreases the disease symptoms and prolongs survival rate of infected mice

**Figure 8:**
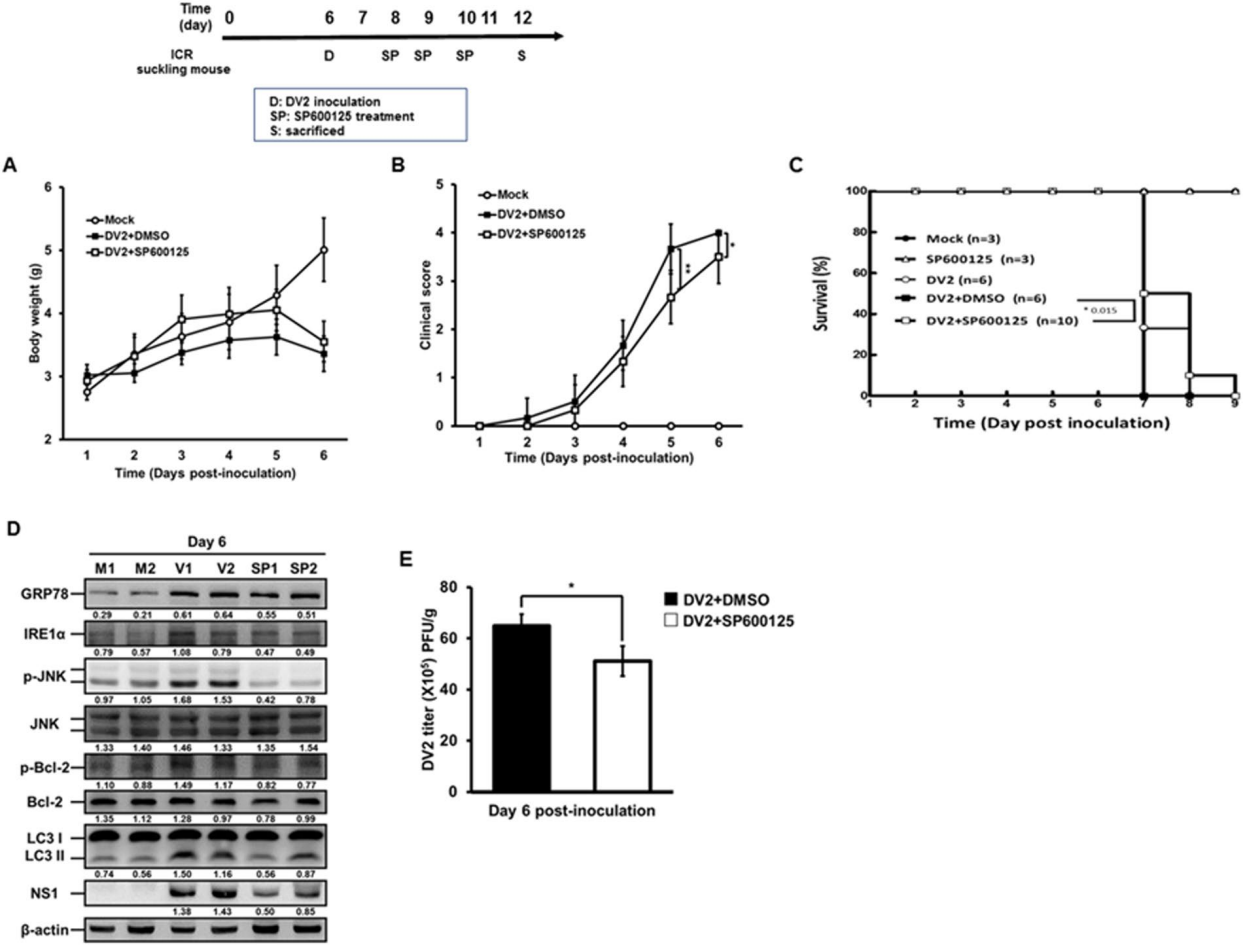
Inhibition of phosphorylated JNK alleviated the disease symptoms and viral titer in DENV2 infected ICR suckling mice. Six-day-old ICR suckling mice (Mock: n = 6, DENV2 + DMSO: n = 6, and DENV2 + SP600125 n = 6) were i.c. treated with SP600125 (0.1 mM; 3 μl/g) at day 2, 3, 4 after inoculation of DENV2 (2.5 × 10^5^ PFU/mice). The (**A**) body weight, (**B**) disease symptoms, and (**C**) survival rate were monitored every day after inoculation. (**D**) The brain tissues were harvested and total protein lysate were collected at day 6 p.i. and the expression of GRP78, p-JNK, JNK, LC3-II/ LC3-I and NS4B was analyzed by Western blotting. The number under the band is the quantification of band intensity after normalization with β-actin. SP: mice group was treated with SP600125 after inoculation. (**E**) The viral titer in the mice brain was determined by plaque assay. Significance was defined as *p < 0.05; **p < 0.01 and ***p < 0.001.

Above data demonstrate that IRE-1α-JNK signaling pathway is at the up-stream of DENV-induced autophagy and can regulate viral replication. To further confirm this speculation, initially DENV2-infected neuron SK-N-SH cells were treated with or without JNK inhibitor SP600125. Our data showed that the level of p-JNK (activation of JNK) but not total JNK protein decreased together with decreased p-Bcl-2, LC3-II and DENV2 NS1 proteins. This result indicates that activated JNK is required for DENV2-induced autophagy as well as viral replication in the neuron SK-H-SH cells (S5 appendix, lane 3 vs. lane 2). The ICR suckling mice were i.c. inoculated with DENV2 at day 6 followed by JNK inhibitor SP600125 inoculation at day 8, 9 and 10 (Fig. 8, the experimental design). Our data reveal that the declined body weight at day 6 post-inoculation as well as delayed the progression of clinical symptoms at day 5 and 6 p.i. and prolonged the survival time of the mice in DENV2 infection plus SP600125 treatment group compared to Mock and DENV2 infection only groups (Fig. 8A,B and C). Blocking of JNK signaling *in vivo* with SP600125 treatment reduced the autophagic activity and viral protein expression demonstrated by decreased expression of LC3-II as well as NS1 proteins (Fig. 8D). Our plaque assay data showed decrease of viral titer in DENV2 infection plus SP600125 treatment group compared to DENV2 infection only group (Fig. 8E), suggesting that JNK signaling is involved in autophagic activation and plays a partial role in DENV2 viral replication *in vivo*. In conclusion, our findings imply that SP600125 reduces autophagy activity through the inhibition of phosphorylated JNK to decrease viral load, and consequently alleviates the clinical symptoms of DENV2 infection and prolonged the survival of the infected mice.

## Discussion

In this study, we explored the effect of DENV2 infection on ER stress associated UPRs and their relationship with autophagy, viral load and pathogenesis both *in vitro* and *in vivo*. We reveal that during DENV infection, among the three ER stress associated UPRs, IRE1α-JNK is the major signaling pathway to induce Bcl-2 phosphorylation and cause dissociation of the BECN1-Bcl-2 complex to release Beclin1. Released Beclin1 then triggers autophagic activity. In addition, DENV induced IRE1α-XBP-1 signaling pathway increases cytokine production of IL-8 and TNFα.

Datan *et al*. reported that PERK signaling pathway participates in DENV-mediated autophagy induction and viral replication in dog MDCK and mouse MEF cells^39^, however, what UPRs are involved in DENV infected human hepatoma and lung cancer cells remains unclear. Similarly, we showed that only live DENV2 infection could induce ER stress and autophagic activity in human hepatoma cells (Fig. 1). UPR signaling induces ER membrane expansion through the development of ER sheets driven by lipid biosynthesis, for DENV2 replication produces large amount viral-related materials accumulated in ER lumen to induce ER stress. Extension of ER membrane alleviates ER stress and prevents cell death^44^. Upon infection, DENV2 NS3 and NS4A proteins trigger ER membrane rearrangement and expansion at the early stage of infection in a UPR independent manner^4^. Furthermore, disrupted ER membrane functions as the platform for DENV2 replication, and this phenomenon often refers as paracrystalline arrays or vesicle packets^4,45^. We have reported that DENV2-induced autophagy promotes viral production by forming the autophagosome, which acts as the dock for viral replication complex^23^. Welsch *et al*. reported that DENV modifies ER membrane structure to pack viral genome and to promote its replication^45^. Moreover, DENV2-induced autophagosome recruits host triglycerides to increase β-oxidation activity which enhances ATP production for viral replication^25,46^. DENV capsid protein subverts lipid droplets derived from ER membrane for viral particle formation^47^. Taken together, these findings indicate that autophagy not only provides the site and the energy for virus replication but also promotes host cell survival, which further sustains viral production. Similarly, other members of *Flaviviriuses* HCV, JEV, and WNV utilize host lipid system to enhance viral replication^26,48–51^. Therefore, we hypothesize that at the early stage of DENV2 infection; viral proteins may alter ER membrane and induce mild autophagic activity. Subsequently, continuous DENV2 infection produces large amount of viral proteins that accumulate in ER lumen to induce prolonged ER stress. However, DENV2 can suppress ER stress-triggered cell death by modulating UPRs to further promote autophagic activity^39^, which provides the replication platform and ATP energy for viral production.

The origin of DENV-induced autophagosome membrane remains unclear. Tooze and Yoshimori reported that the phagophore (also called the isolation membrane) is synthesized from the membrane of Golgi, ER, or mitochondria^52,53^, which progresses to form autophagosomes. A tomography study reported that DENV replicates on the ER cisternae invaginations not on the classical autophagosome under the cryo-EM^45^. Nevertheless, whether viral particle is packed in the autophagosome during infection needs further investigation.

Previous study reported that polyglutamine 72 repeat aggregates-induced ER stress increases ATG12 mRNA and protein via phosphorylated eIF2α, followed by conversion of LC3 type I to type II which represents autophagosome formation^8^. However, others reports claimed that autophagy induced by ER stress, starvation or viral infection, the ATF4-ATG12 expression signaling pathway is involved but is eIF2α dispensable^54^. Therefore, the role of eIF2α in regulation of autophagy remains contradictory. One report showed that DENV2 induces eIF2α phosphorylation at the early stage of infection, but this event is rapidly reversed at the later time of DENV infection to assist viral protein synthesis^15^. It is known that unphosphorylated eIF2α mediates the binding of tRNA^met^ to the ribosome in a GTP-dependent manner to initiate translation^55^. Because eIF2α is essential for translation, the possibility that knockdown of eIF2α leads to UPR-independent inhibition of autophagic activity and DENV2 replication (Fig. 3A) cannot be excluded.

The level of the conjugated form of ATG5-ATG12 is significantly increased under the treatment of DENV antibody-dependent enhancement (ADE) complexes in the human monocyte cells^56^. Differently, the level of ATG5-ATG12 conjugated form decreased from 12 h to 24 h p.i. in DENV2- infected monocyte cell under non-ADE conditions^56,57^. We have demonstrated that DENV2 infection could induce autophagic flux^23^, which progresses in a dynamical fashion during infection (unpublished data). It is probable that the excessive autophagic activity causes cell death, and is unfavorable for DENV infection. Therefore, proper modulation of autophagic level maintains cell viability which is beneficial for viral replication. In addition, eIF2α-ATF4-CHOP signaling pathway induces autophagy regulators ATG6 and ATG8, which reduce ROS level and delay cell death^58^.

The effect of IRE1α-XBP1 signaling pathway on autophagy activity has been reported. Deficiency of XBP1 prompts autophagic activity^59–61^. In contrast, another study reported that knockdown of XBP1 decreases autophagy activity^62^. Differently, we found that silence of XBP1 has no effect on autophagic activity and DENV2 replication (Fig. 4C). Moreover, others reported that XBP1 participates in regulating immune responses, including differentiation of effector CD8^+^ T cells during acute infection^63^. XBP1 also correlates with human inflammatory bowel disease (IBD) and Crohn’s disease. XBP1 deficiency causes spontaneous inflammation of the gastrointestinal tract due to lack of effective levels of antimicrobial agents. Furthermore, the XBP1 knockout mice (XBP1^−/−^) showed reduction in microbial killing, increase of susceptibility to dextran sodium sulfate-induced colitis. These mice also showed high expression level of tumor necrosis factor alpha (TNFα) in colonic mucosa^64,65^. Surprisingly, our data showed that suppression of XBP1 by sh-RNA reduced the mRNA levels of pro-inflammatory cytokines IL-8 and TNFα in DENV2-infected Huh7 cells (S3 appendix), suggesting that XBP1 participates in DENV2-related immune and inflammation responses.

JNK-related signaling pathway participates in induction of apoptosis and autophagy^11^. Similarly, we demonstrate that phosphorylated JNK under DENV2 infection could trigger Bcl-2 phosphorylation and its dissociation from BECN1, which is responsible for autophagy activation (Fig. 6B and C). In addition, phosphorylation of JNK and activation of the downstream activating protein 1 (AP-1) regulate various cytokines, including IL-6, IL-8, TNFα and monocyte chemotactic protein-1 (MCP-1)^66–72^. Here, we suggest that UPR related signaling pathways are responsible for both autophagy induction and cytokine production during DENV2 infection.

**Figure 9:**
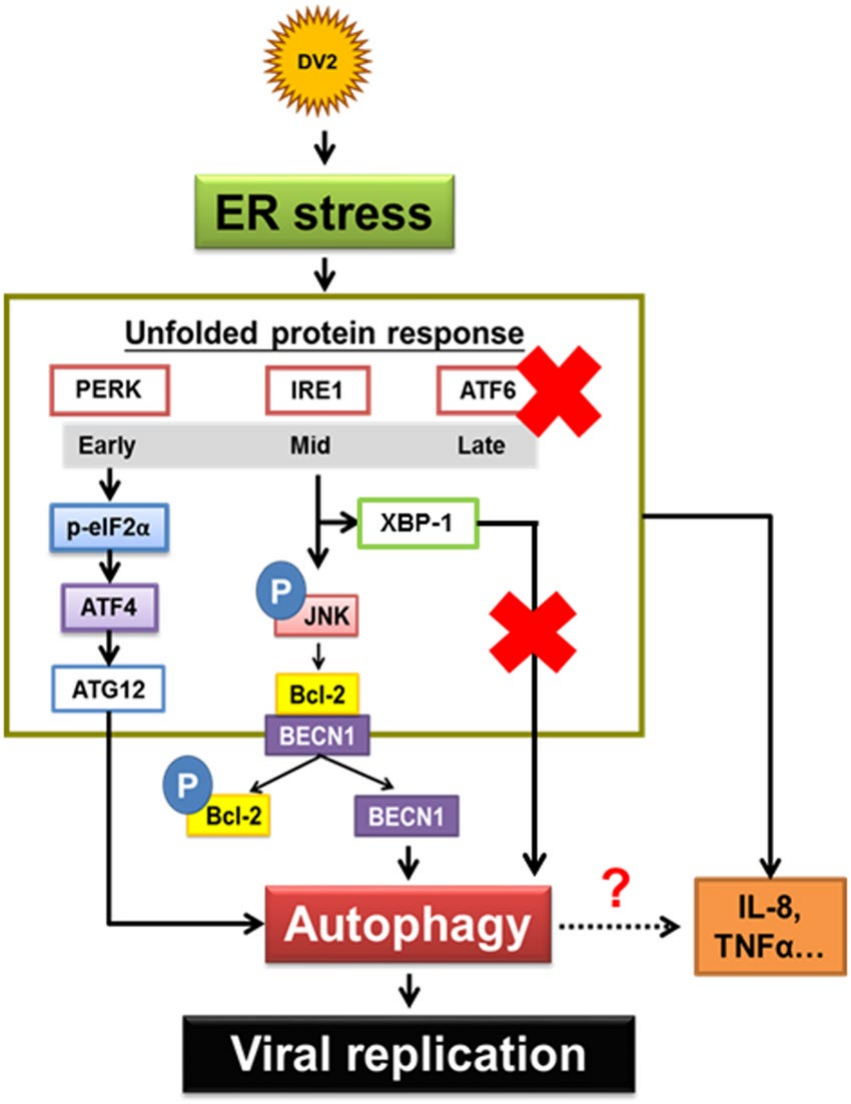
DENV2-induced autophagy is ER stress-UPR dependent, involving PERK-eIF2α-ATF4 and IRE1α-JNK-BECN1 pathways. DENV2 infection temporally modulates UPR in the order of PERK, IRE1 and ATF6 in human cells. PERK-ATF4-ATG12 and IRE1α-JNK are the two major signaling pathways participating in DENV2-induced autophagy and viral replication. The phosphorylated JNK further activates the downstream molecule Bcl-2, and BECN1 then dissociates from Bcl-2/BECN1 complex when Bcl-2 was activated, which is one of the molecules responsible for autophagy induction and promotes viral replication. However, IRE1α-XBP1 pathway shows no impact on DENV2-induced autophagy and viral replication but involved in regulating IL-8 and TNFα production during DENV2 infection. Significance was defined as *p < 0.05; **p < 0.01 and ***p < 0.001.

In conclusion, this study reveals the role of ER stress in induction of DENV2-mediated autophagy and the underlying mechanism, and this event further contributes to viral replication and affects the severity of pathogenesis both *in vitro* and *in vivo*. Furthermore, targeting the molecules in ER stress and autophagy may become a potential treatment for dengue virus infected patients (Fig. 9).

## Materials and Methods

### Cell culture and dengue virus

Huh7 (human hepatoma cell line), A549 (human lung carcinoma cell line), BHK (Baby hamster kidney cell line), MEF (mouse embryo-fibroblast)-ATG5 wild type (*ATG5* ^+/+^), and knockout cells (*ATG5* ^−/−^) were cultured in DMEM (GIBCO, Gaithersburg, MD, USA) supplemented with 10% FBS (Biological Industries, Kibbutz Beit Haemek, Israel), at 37 °C in a 5% CO_2_ incubator. C6/36 (mosquito cell line) cells were maintained at 28 °C in the same culture medium as above cell lines. Trypsin (Sigma, Aldrich, MO, USA) was used to re-suspend the cells. DENV2 (PL0146 strain was isolated from Taiwan) were cultured in C6/36 cell with DMEM containing 2% FBS and incubated at 28 °C for four to five days. The supernatant was collected, filtrated through the 0.22 μm filter, and stored at a −70 °C freezer. The viral titer was determined by plaque assay.

In the following viral experiments, cells were infected with DENV2 at a multiplicity of infection (MOI) of 10 at 37 °C for 2 h, and then the medium was replaced with DMEM containing 10% FBS with or without specific treatments.

### Plaque assay

BHK cells (5 × 10^4^ cells/well) were seeded in the 24-well plate and maintained in 10% FBS/DMEM for overnight. The cells were infected with 150 μl of 2% FBS/DMEM containing serial diluted virus solution. After adsorption at 37 °C for 2 h, the virus solution was replaced with DMEM containing 2% FBS and 0.8% methylcellulose (Sigma). Five days post infection, the methylcellulose medium was removed and the cells were rinsed with 1 × phosphate buffered saline (PBS) twice. After washing, the cells were fixed and stained with 2% crystal violet (Sigma, C6158) solution at room temperature (RT) for overnight. Finally, the crystal violet solution was washed out with D.D water, and the viral titer was determined by plaque assay.

### Western blotting

Protein samples were collected from cultured cells or animal tissues in fresh-prepared RIPA lysis buffer (0.5 M Na_3_VO_4_, 0.5 M EDTA, 0.1 M EGTA, 1 mg/ml leupeptin, 1 mg/ml aprotinin and 0.1 M PMSF) and centrifuged with 13600 rpm at 4 °C for 20 min. The concentration of each sample was determined and loaded into a sodium dodecyl sulfate-polyacrylamide gel (SDS-PAGE) followed by electrophoresis and transferred to a PVDF membrane (Millipore, Billerica, MA, USA). The membrane was soaked in 5% skim milk or 5% BSA (Sigma) with TBST buffer (0.02 M Tris-base, 0.15 M NaCl, 0.1% Tween 20, pH 7.4) and blocked at RT for 1 h. The following antibodies were used: monoclonal antibodies for NS1 (a gift of Dr. H.Y. Lei, National Cheng Kung University); phospho-eIF2α (Cell signaling technology, Beverly, MA, USA); eIF2α (Cell signaling technology); IRE1α (Cell signaling technology); phospho-SAPK/JNK (Cell signaling technology); JNK (Cell signaling technology); BECN1 (Santa Cruz Biotechnology, CA, USA); phospho-Bcl-2 (Cell signaling technology); Bcl-2 (Abcam, Cambridge, United Kingdom); ATF6α (Santa Cruz Biotechnology); LC3 (MBL, Nagoya, Japan); β-actin (Sigma) and incubated at 4 °C for overnight. After washing with TBST buffer three times for 30 min, the membrane was incubated with secondary antibodies at RT for 1 h. Finally, the membrane was rinsed with enhanced chemiluminescence (ECL) (Millipore) and exposed by BioSpectrum AC (101-206-009; UVP, Upland, CA, USA). The results of Western blotting were quantified by ImageJ software (National Institutes of Health, Bethesda, MD, USA).

### Immunoprecipitation (IP)

Huh7 cells were infected with DENV2 and the protein were collected with RIPA buffer at 36 h p.i. Cellular protein (1 mg) was mixed with RIPA buffer-washed protein G agarose bead (50 μl/ sample; GE Healthcare, United Kingdom) to remove nonspecific binding protein, and supernatant were collected after centrifugation and incubated with specific antibodies at 4 °C for overnight. After washing with RIPA buffer, the protein G agarose bead (60 μl/ sample) was mixed with the samples to pull-down the immunocomplex after centrifugation. Western blot analysis was conducted and followed by hybridization with anti-BECN1 (1:1000 dilution) or anti-Bcl-2 (1:1000 dilution) antibody.

### Transient transfection

Huh7 cells (3 × 10^5^ cells/ well) were plated into the 6-well plate, and the transfection was performed following the manufacturer’s instructions of Turbofect transfection kit (Fermentas, Carlsbad, CA, USA). The transfected Huh7 cells were infected with DENV2 at a MOI of 10 at 18 h post transfection at 37 °C for 2 h and replaced with 10% FBS DMEM. The samples were collected at 36 h p.i. for different experiments.

### PCR and RT-PCR

For reverse transcription polymerase chain reaction (RT-PCR), the complementary DNA (cDNA) was synthesized from 2 μg of total RNA using High-Capacity cDNA Reverse Transcription Kits (Applied Biosystem, California, USA). Transcription procedure was based on the modified manufacturer protocols by mixing 10 μl of 2 μg RNA and 10 μl of 2 × RT Master Mix. The thermal cycler program was 25 °C 10 min, 37 °C 120 min, 85 °C 5 min and 4 °C until the end of reaction. The sequences of PCR primers were used as follows:

NS1 forward 5′-ATGGATCCGATAGTGGTTGCGTTGTGA-3′

NS1 reverse 5′-ATCTCGAGGGCTGTGACCAAGGAGTT-3′;

XBP1 forward 5′-CCTTGTAGTTGAGAACCAGG-3′

XBP1 reverse 5′-GGGGCTTGGTATATATGTGG-3′;

IL-8 forward 5′-AAGAGAGCTCTGTCTGGACC-3′

IL-8 reverse 5′-GATATTCTCTTGGCCCTTGG-3′;

TNFα forward 5′-AGGCAGGTTCTCTTCCTCTCAC-3′

TNFα reverse 5′-TGATTAGAGAGAGGTCCCTGGG-3′;

β-actin forward 5′-GGCGGCACCACCATGTACCCT-3′

β-actin reverse 5′-AGGGGCCGGACTCGTCATACT-3′

The PCR protocol was conducted together with the above primer pairs at 94 °C 5 min, 94 °C 30 sec, 55 °C 30 sec, 72 °C 1 min (30 cycles), 72 °C 5 min and 4 °C until the end of reaction.

### Immunofluorescent assay (IFA)

Huh7 cells were plated on a cover glass in the 6-well plate (1 × 10^5^ cells/well) and incubated at 37 °C overnight. The cells were infected by DENV2 at a MOI of 10 for 2 h and then replaced with 10% FBS/DMEM. The sample was collected at 36 h p.i. and fixed with 3.7% paraformaldehyde (Merk, Darmstadt, Germany) at RT for 30 min. Cells were rinsed with PBS and perforated with 0.1% Triton-X-100 (Merk) at RT for 30 min. After washing, cells were blocked with 2% BSA at RT for 1 h, and were incubated at 4 °C overnight with the primary antibody as following: NS1 (Abcam); Bcl-2 (Abcam); BECN1 (Abcam). The cells were washed with PBS and incubated with the secondary antibody (in the dark at RT for 1 h) as following: FITC-conjugated antibody (Jackson Laboratories); PE-conjugated antibody (Jackson Laboratories). Cellular nucleus were strained by Hochest 33258 (Sigma), and incubated in dark at RT for 30 min. Finally, the samples were washed with PBS and the fluorescent signal was determined with fluorescence microscope (Olympus IX71, Tokyo, Japan) or multi-photon confocal microscope (Olympus FluoView 1000MPE).

### Animal experiment

Six-day-old ICR suckling mice were obtained from the Laboratory Animal Center of National Cheng Kung University, Tainan, Taiwan. The experimental protocol complied with Taiwan′s Animal Protection Act and was approved by the Laboratory Animal Care and Use Committee of the National Cheng Kung University (IACUC approval No. 103023). The suckling mice were randomized into indicated groups, and DENV2 (2.5 × 10^5^ PFU/mice) was intracranially injected into six-day-old ICR suckling mice, which were sacrificed at the 5^th^ day post inoculation. The brain tissues were aseptically collected, weight and homogenized in 2% FBS/DMEM (1 ml). The supernatant was collected by centrifugation under 4 °C, 8000 rpm for 15 min and frozen at −70 °C for plaque assay study. Tissue samples were homogenized with RIPA buffer (1 ml). The supernatant was collected by centrifugation under 4 °C, 13600 rpm for 20 min and frozen at −70 °C for western blotting analysis.

### Statistical analysis

Data are presented as the mean ± SD. Differences between the test and control groups were analyzed using the Student′s *t* test. Significance was set at P < 0.05^*^; P < 0.01^**^; P < 0.001^***^.

## Electronic supplementary material

Supplementary Data
