# Supplementary material for: Dengue virus-induced ER stress is required for autophagy activation, viral replication, and pathogenesis both in vitro and in vivo
Source: Sci Rep. 2018 Jan 11;8:489. doi: 10.1038/s41598-017-18909-3 (PMC5765116; doi:10.1038/s41598-017-18909-3)
Supplement: Supplementary file 1 — Supplementary Data [file 41598_2017_18909_MOESM1_ESM.pdf]

Dengue virus-induced ER stress is required for autophagy activation, viral replication, and pathogenesis both *in vitro* and *in vivo*

Ying-Ray Lee[1#], Szu-Han Kuo[2#], Ching-Yen Lin[1], Po-Jung Fu[2], Yee-Shin Lin[2], Trai-Ming Yeh[3], and Hsiao-Sheng Liu[2*]

[1]Department of Medical Research, Chiayi Christian Hospital, 600 Chiayi, Taiwan. (Ying-Ray Lee: yingray.lee@gmail.com; Ching-Yen Lin: jouyuan22@gmail.com)
[2]Department of Microbiology and Immunology, College of Medicine, National Cheng Kung University, 701 Tainan, Taiwan. (Szu-Han Kuo: s46004022@mail.ncku.edu.tw; Po-Jung Fu: U9807038@cmu.edu.tw; Yee-Shin Lin: yslin1@mail.ncku.edu.tw; Hsiao-Sheng Liu: a713@mail.ncku.edu.tw)
[3] Department of Medical Laboratory, Science and Biotechnology, College of Medicine, National Cheng Kung University, 701 Tainan, Taiwan. (Trai-Ming Yeh: today@mail.ncku.edu.tw)

Running title: Dengue virus induces ER stress, autophagy, viral replication and pathogenesis

Key Words: Dengue virus; ER stress; Unfolded protein response; Autophagy, viral replication

*Corresponding author: Hsiao-Sheng Liu
Department of Microbiology and Immunology,
College of Medicine,
National Cheng Kung University,
1 University Road,
Tainan, 70101,
Taiwan.
Telephone number: 886-2353535, ext. 5630.
E-mail: a713@mail.ncku.edu.tw

# These authors contributed equally to this work.

Supplementary Data

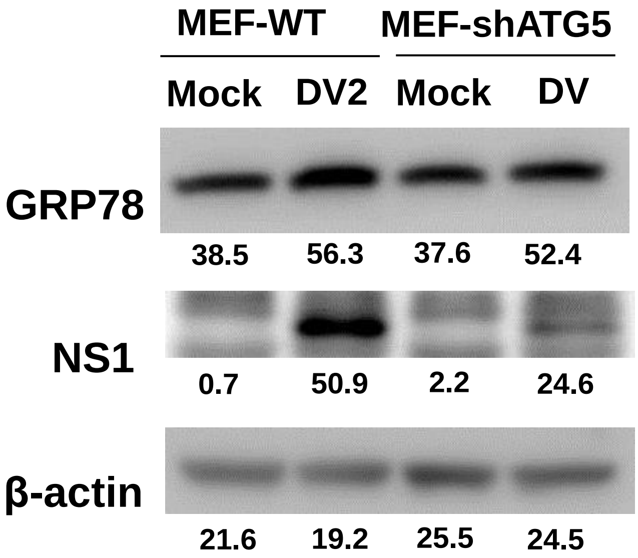

S1 appendix. Autophagy does not affect DENV2-induced ER stress. MEF cells were mock or shATG5 transfected for 18 h, and then with or without DENV2 infection (MOI=20). The protein levels of GRP78 and DENV2-NS1 were investigated by Western blotting using specific antibodies at 24 h p.i.. β-actin was used as the loading control. The number under the band is the quantification of band intensity after normalization with β-actin.

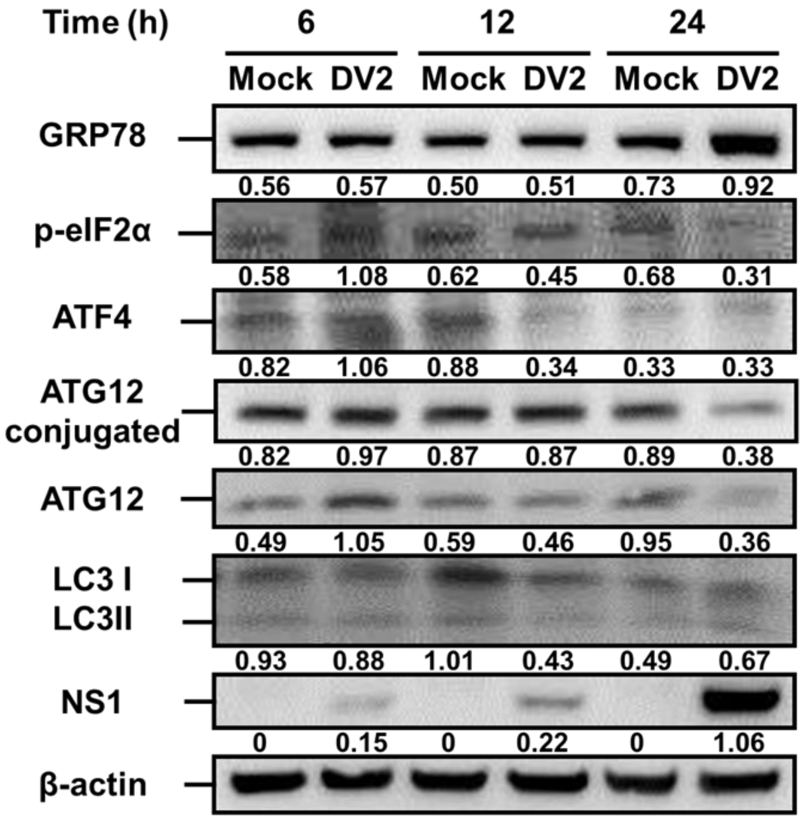

S2 appendix. PERK-eIF2α-ATF4-ATG12 signaling pathway is involved in DENV2-induced autophagy activation. Huh7 cells were infected with DENV2 (MOI=10) and the samples were collected at 6 h, 12 h and 24 h p.i.. The protein levels of GRP78, p-eIF2α, ATF4, ATG12 free form and conjugated form, LC3 II/LC3 I and NS1 were detected by Western blotting at the indicated time points. The number under the band is the quantification of band intensity after normalization with β-actin.

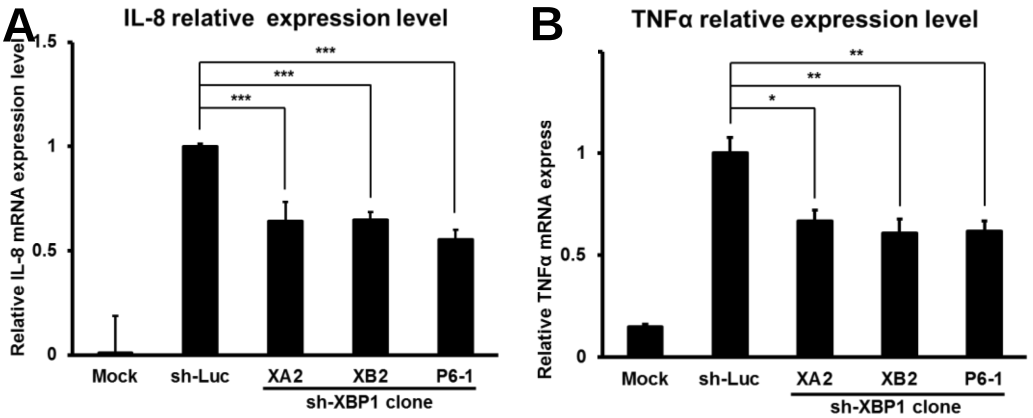

S3 appendix. Silencing of XBP1 reduces IL-8 and TNFα mRNA expression during DENV2 infection. Huh7 cells were transfected with sh-XBP1 and the cells were infected with DENV2 (MOI=10). The mRNA expressions of (A) IL-8 and (B) TNFα were determined by real-time PCR after 36 h p.i.. Expression of β-actin mRNA was used as an internal control, and the relative levels were quantification after normalization with β-actin.

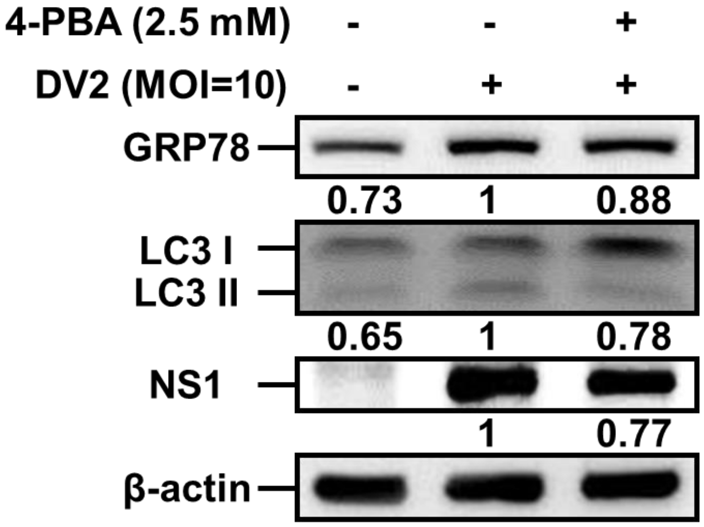

S4 appendix. ER stress inhibitor suppressed autophagic activity and DENV2 NS1 protein expression in SK-N-SH cells. SK-N-SH cells were infected with DENV2 (MOI=10) with or without the treatment of 4-PBA (2.5 mM), and the expressions of GRP78, LC3 and DENV2-NS1 proteins were determined by Western blotting at 36 h p.i. using specific antibodies.

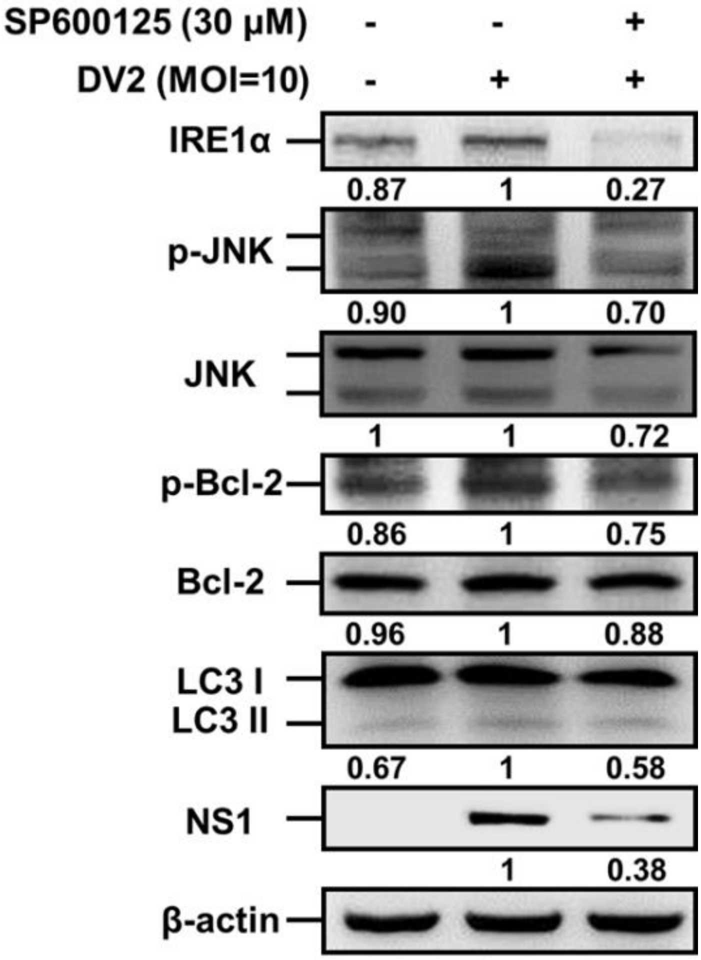

S5 appendix. The inhibitor of JNK signaling pathway suppressed autophagic activity and DENV2 NS1 protein expression in SK-N-SH cells. SK-N-SH cells were infected with DENV2 (MOI=10) with or without the treatment of SP600125 (30 μM), the expressions of IRE1α, p-JNK, LC3 and NS1 proteins were determined with Western blotting at 36 h p.i..
